# The Relationship between Maternal Nutrition during Pregnancy and Offspring Kidney Structure and Function in Humans: A Systematic Review

**DOI:** 10.3390/nu10020241

**Published:** 2018-02-21

**Authors:** Yu Qi Lee, Clare E. Collins, Adrienne Gordon, Kym M. Rae, Kirsty G. Pringle

**Affiliations:** 1 School of Biomedical Sciences and Pharmacy, Faculty of Health and Medicine, University of Newcastle, Newcastle, NSW 2308, Australia; yuqi.lee@uon.edu.au; 2 Priority Research Centre for Reproductive Sciences, University of Newcastle, Newcastle, NSW 2308, Australia; kym.rae@newcastle.edu.au; 3 School of Health Sciences, Faculty of Health and Medicine, University of Newcastle, Newcastle, NSW 2308, Australia; clare.collins@newcastle.edu.au; 4 Priority Research Centre in Physical Activity and Nutrition, University of Newcastle, Newcastle, NSW 2308, Australia; 5 Charles Perkins Centre, University of Sydney, Camperdown, NSW 2006, Australia; adrienne.gordon@sydney.edu.au; 6 Gomeroi Gaaynggal Centre, Faculty of Health and Medicine, University of Newcastle, Tamworth, NSW 2340, Australia; 7 Department of Rural Health, School of Medicine and Public Health, Faculty of Health and Medicine, University of Newcastle, Tamworth, NSW 2340, Australia; 8 Priority Research Centre for Generational Health and Ageing, University of Newcastle, Newcastle, NSW 2308, Australia

**Keywords:** pregnancy, nutrition, obesity, diabetes, kidney disease

## Abstract

The intrauterine environment is critical for fetal growth and organ development. Evidence from animal models indicates that the developing kidney is vulnerable to suboptimal maternal nutrition and changes in health status. However, evidence from human studies are yet to be synthesised. Therefore, the aim of the current study was to systematically review current research on the relationship between maternal nutrition during pregnancy and offspring kidney structure and function in humans. A search of five databases identified 9501 articles, of which three experimental and seven observational studies met the inclusion criteria. Nutrients reviewed to date included vitamin A (*n* = 3), folate and vitamin B12 (*n* = 2), iron (*n* = 1), vitamin D (*n* = 1), total energy (*n* = 2) and protein (*n* = 1). Seven studies were assessed as being of “positive” and three of “neutral” quality. A variety of populations were studied, with limited studies investigating maternal nutrition during pregnancy, while measurements of offspring kidney outcomes were diverse across studies. There was a lack of consistency in the timing of follow-up for offspring kidney structure and/or function assessments, thus limiting comparability between studies. Deficiencies in maternal folate, vitamin A, and total energy during pregnancy were associated with detrimental impacts on kidney structure and function, measured by kidney volume, proteinuria, eGFR_cystC_ and mean creatinine clearance in the offspring. Additional experimental and longitudinal prospective studies are warranted to confirm this relationship, especially in Indigenous populations where the risk of renal disease is greater.

## 1. Introduction

In 1989, David Barker proposed that exposure to environmental stimuli or insults during critical phases of intrauterine development may induce compensatory responses in the fetus that permanently alter offspring phenotype at birth [1,2]. This phenomenon has since been termed the Developmental Origins of Health and Disease (DOHaD) hypothesis [3]. Maternal insults during intrauterine development (e.g., poor nutrition, severe stress or illness) impact on offspring risk for various health conditions including cardiovascular disease (CVD), hypertension, obesity, type 2 diabetes and metabolic syndrome [4], as well as chronic kidney disease (CKD) and end-stage renal disease (ESRD) in later life.

In a recent review, numerous studies in animals have demonstrated that maternal over or under nutrition can lead to altered nephrogenesis, with a permanent reduction in nephron endowment and detrimental consequences for offspring renal health [5]. The most common nutrition intervention in animal studies examines maternal protein restriction, which was associated with a reduction in nephron number and hypertension in adult offspring. Woods et al. demonstrated that Sprague–Dawley rats fed a low protein diet (8.5% protein; control animals on normal chow = 19% protein) throughout pregnancy delivered male offspring with a reduced number of nephrons [6,7]. Langley-Evans et al. demonstrated that male and female Wistar–Kyoto rat offspring had fewer nephrons when mothers were fed a low protein diet (9% casein; control animals = 18% casein) throughout gestation, or only in mid and late gestation [8]. However, no reduction in nephron number occurred when mothers were exposed to a low protein diet in early gestation [8].

Likewise, there is evidence in animal models that maternal micronutrient deficiency can have detrimental effects on kidney development. Lelievre-Pegorier et al. reported on a rat model, where mothers were fed a vitamin A-deficient diet for three weeks prior to conception until day 21 of gestation, that there was a 20% reduction in the number of nephrons compared to controls [9]. Maternal iron deficiency was associated with lower glomerular number and higher systolic blood pressure in adult rat offspring compared to control rats [10].

Although the majority of studies examining the effects of nutrition on fetal programming of renal disease focus on maternal undernutrition, it is apparent that maternal over-nutrition and obesity in pregnancy also increases the risk of suboptimal offspring health outcomes. Researchers have attempted to model the effects of maternal obesity, mostly by feeding a high-fat, high-fructose ‘Western’ diet to pregnant rats [11,12]. This was typically associated with development of metabolic syndrome and renal injury in adult offspring [13,14,15,16,17,18]. Male Sprague–Dawley rat offspring exposed to a high fat/high fructose maternal diet in utero and fed a high-fat/high-fructose diet neonatally had increased albuminuria (450%) and glomerulosclerosis (190%) and weighed ∼23% more than offspring born to mothers fed a standard rat chow diet, regardless of their neonatal diet [18]. These results support the hypothesis that over-nutrition in utero also has a detrimental impact on later renal health in offspring.

While there is convincing evidence from animal studies of a link between maternal nutrition during pregnancy and kidney structure and function in the offspring, evidence from human studies is yet to be critically appraised and synthesised. To our knowledge, a systematic review of human studies has not yet been performed. Given the considerable evidence from animal studies and that the origins of many chronic diseases can be traced back to in utero conditions, it is an important issue to systematically review. During pregnancy, a mother’s health and dietary habits can have a major impact on the development and future well-being of the infant [19,20]. Understanding the relationship between maternal nutrition during human pregnancy and offspring kidney structure and function could therefore inform the development of future interventions aimed at improving maternal health and optimising infant renal health.

Therefore, the objective of this study was to systematically review the best available evidence on the relationship between maternal nutrition during pregnancy and offspring kidney structure and function. This review considered two main questions:

1. What is known about the relationship between maternal nutrition during pregnancy and offspring kidney structure and function?
2. Which areas of research have not been addressed in the current literature?

## 2. Methods

The systematic review protocol was registered with the online Prospero database (CRD42016047758) [21] and adhered to the Preferred Reporting Items for Systematic Reviews and Meta-analysis (PRISMA) statement [22].

### 2.1. Study Identification

Studies published in English prior to November 2017 from five relevant databases (CINAHL, Cochrane, EMBASE, MEDLINE, Scopus) were collected using identified keywords and index terms. The search terms were divided into three groups: (1) Pregnancy/ or pregnan*.mp., Maternal, Mother*, Prenatal.mp. and (2) nutrition.mp., dietary habits.mp. or Food Habits/, food intake.mp. or Eating/, diet.mp. or Diet/, dietary supplements.mp. or Dietary Supplements/, undernutrition.mp. or Malnutrition/, overnutrition.mp. or Overnutrition/ or Nutritional Physiological Phenomena/, diabetes.mp. or Diabetes, Gestational/, Hyperglycemia, Obesity/ or obesity.mp., Weight Gain.mp. or Weight Gain/, Overweight and (3) kidney.mp. or Kidney/, nephron.mp. or Nephrons/, renal.mp. The Boolean phrase AND was used between groups and OR within groups. This paper focuses on the literature which investigated the association between maternal nutrition during pregnancy and offspring kidney structure and function in humans. The other aspects of maternal intrauterine environment (obesogenic or elevated plasma glucose) and their association with offspring kidney outcomes will be reported separately.

### 2.2. Inclusion Criteria

#### 2.2.1. Types of Participants

This current review only considered human studies and all participants had to be pregnant (at any stage of gestation) at the time of the study.

#### 2.2.2. Types of Studies

Study types included were experimental studies, including pre-post, pseudo-randomised controlled trials and randomised controlled trials. Observational studies, including cross-sectional studies, case-control studies and prospective and retrospective cohort studies were also considered.

#### 2.2.3. Types of Exposures/Interventions

The type of exposure considered for this current review was maternal nutrition during pregnancy.

#### 2.2.4. Types of Outcome Measures

Studies were considered for inclusion if they included a measure of offspring kidney function and/or structure as a primary or secondary outcome. Kidney structure outcome measures in offspring were: kidney volume, kidney size, kidney mass, kidney structure, glomerular size and nephron endowment. Outcomes related to kidney function in offspring included: proteinuria, urinary albumin/creatinine, microalbuminuria, albuminuria, glomerular filtration rate, urinary protein/creatinine, urinary nephrin/creatinine and urinary sodium/potassium.

### 2.3. Study Selection

All studies identified were retrieved and exported to the reference management system EndNote (Version X8, Thomson Reuters, New York, NY, USA). The first phase of study identification included an assessment of study inclusion based upon the title, abstract and description/MESH headings. All stages were conducted by two independent reviewers. Full texts were retrieved for those papers that met the inclusion criteria from the title and abstract. The full article was retrieved for review and data extraction. When in disagreement at any step, a third independent reviewer was consulted to make a decision.

### 2.4. Study Quality

All included studies were assessed for methodological quality using the American Dietetic Association (ADA) Quality Criteria Checklist [23], which consisted of ten criteria to assess the strength of the research design, relevance and validity. The items assessed included: the method of sample selection, methods of controlling for confounding factors, reliability of outcome measures and statistical analysis. Using this checklist, two independent reviewers rated the overall quality of the studies as positive, neutral or negative. A third reviewer was consulted when needed. No studies were excluded based on quality ratings.

### 2.5. Data Extraction and Synthesis

Data extraction was conducted by one reviewer and cross-checked by a second independent reviewer for accuracy and consistency. Participant information, study design and intervention characteristics as well as data related to review outcomes were extracted. Meta-analysis of data was not expected to be possible due to heterogeneity in maternal exposures during pregnancy and measures of kidney outcome and function. Therefore, the effect of maternal nutrition during pregnancy on offspring kidney structure and function was described in a narrative synthesis. A structured summary, direction of effect, the strength of the evidence for the effect, and whether this was consistent across studies were highlighted in the data synthesis.

## 3. Results

### 3.1. Study Selection

**Figure 1:**
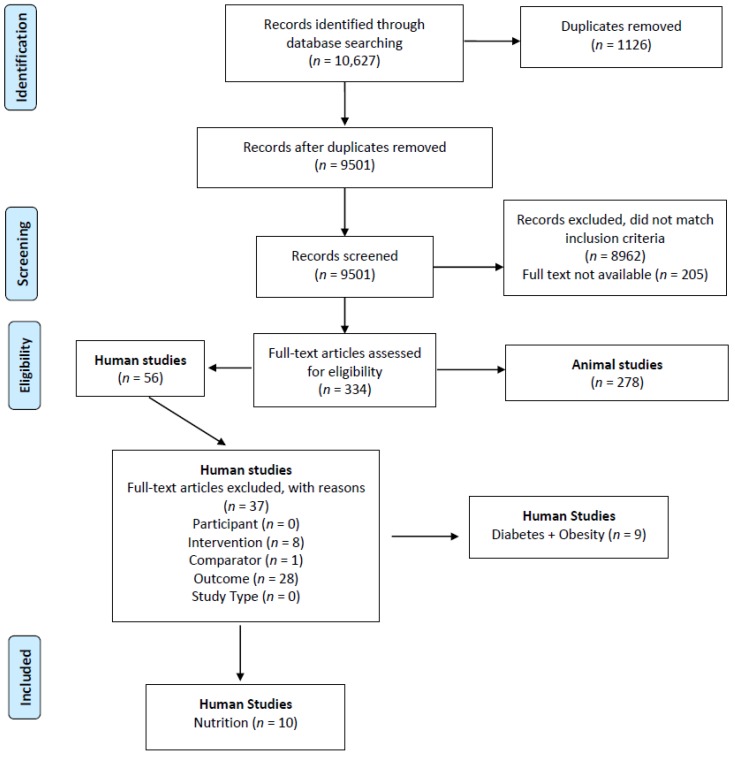
Preferred Reporting Items for Systematic Reviews and Meta-analysis (PRISMA) flow diagram of the study selection process.

Figure 1 illustrates that the initial database search identified 9501 articles after removal of duplicates, with 8962 records excluded following title and abstract review for eligibility. Despite online inter-library searches, full texts were unavailable for 205 records. Of the 334 full-text articles retrieved, 278 articles were animal studies, with 56 articles on humans. After screening the human studies based on the eligibility criteria, 37 were excluded. The primary reasons for exclusion of full-text papers were outcomes not related to offspring kidney structure or function (*n* = 28) and exposure/intervention not related to maternal nutrition or obesity or diabetes during pregnancy (*n* = 8). Of the remaining 19 articles, ten articles focused on maternal nutrition during pregnancy and offspring kidney outcomes and were included in this current paper.

### 3.2. Study Characteristics

Included studies (*n* = 10) were published between 2005 and 2017 and were conducted in six countries including Bangladesh (*n* = 1) [24], Nepal (*n* = 2) [25,26], China (*n* = 1) [27], Netherlands (*n* = 4) [28,29,30,31], Canada (*n* = 1) [32] and Egypt (*n* = 1) [33]. The common measures of kidney structure were kidney volume, while kidney function measures were microalbuminuria, glomerular filtration rate (GFR), and albuminuria. The majority of studies included both male and female offspring, only one study examined female offspring exclusively [27]. There was a large range in the age of offspring at the time of assessment, from 3-days old to 50-years of age.

Of the included studies, three were randomized control trials (RCTs) [24,25,26] (Table 1) that evaluated the relationship between maternal nutrition during pregnancy and offspring kidney structure and function. The timing of these interventions (vitamin A and micronutrient supplementation) were pre-pregnancy, during pregnancy and/or up to three months postpartum.

Table 2 describes the characteristics of the observational studies included in this review. Seven observational studies evaluated the relationship between maternal nutrition during pregnancy and offspring kidney structure and function, of which two were retrospective cohort studies [27,28] and five were prospective cohort studies [29,30,31,32,33]. Factors that prevented meta-analysis from being performed included the heterogeneity of outcome measures used to measure kidney structure and function, the large variation in the age at follow-up of offspring, and the diverse components of maternal diets that were assessed.

### 3.3. Risk of Bias of Included Studies

In total, seven studies were classified as of high methodological quality, of which three were RCTs [24,25,26] and four were observational [28,29,30,31]. All three RCTs randomly allocated participants to study groups using appropriate methods, however, none described the randomisation procedure in sufficient detail [24,25,26] and all failed to sufficiently describe the allocation concealment procedure for the randomization process. Inclusion/exclusion criteria and demographics of subjects were clearly described in the four observational studies [28,29,30,31]. The remaining three studies [27,32,33] were rated as of neutral quality. Due to the failure to describe the demographics of subjects [32], it was challenging to ascertain the sample characteristics (e.g., gender, ethnicity, socio-economic status) and how they were assessed, which limited the generalisability and applicability to broader population groups. Confounding factors were also not clearly identified or taken into account in the statistical analysis in these studies [32,33] (Refer to Supplementary Table S1).

### 3.4. Relationship between Maternal Nutrition during Pregnancy and Offspring Kidney Structure or Function (Table 3 and Table 4)

#### 3.4.1. Vitamin A

Three studies focused on maternal vitamin A deficiency during pregnancy but reported mixed results. In a prospective pilot study by Goodyer et al., a group of pregnant women from Bangalore (India) were compared with pregnant women from Montreal (Canada) [32]. The mean combined renal volume/body surface area (mL/m^2^) for offspring at 2–6 weeks of age was significantly lower in Bangalore (vitamin A deficient) compared to Montreal (vitamin A replete) (*p* < 0.01) (Table 4) [32]. A similar finding was reported among Egyptian infants in a prospective study by El-Khashab et al., where a reduction in renal size at birth was associated with lower maternal serum retinol concentration during pregnancy (Table 4) [33]. However, in a population residing in the rural Sarlahi District of Nepal, there was no overall effect of maternal supplementation with vitamin A or *β*-Carotene before, during and after pregnancy on blood pressure or risk of microalbuminuria in offspring at ten years (Table 3) [26].

#### 3.4.2. Folate & Vitamin B_12_

A study among 3524 mothers and their 6–8 year-old children in a rural Sarlahi District of Nepal found all supplementation groups who received folic acid had a significant reduction in the risk of microalbuminuria (≥3.4 mg/mmol albumin/creatinine), when compared with the control group (containing vitamin A only) (Table 3) [25]. Results from a prospective cohort study by Miliku et al. in the Netherlands among 4226 mothers and their six year-old children, indicated no association between folic acid supplement intake and childhood kidney outcomes (combined kidney volume, eGFR_cystC_, eGFR_creat_ and risk of microalbuminuria) (Table 4) [31]. However, the results suggested that higher circulating maternal folate concentrations in early pregnancy were associated with larger childhood combined kidney volumes (*p* < 0.01) (Table 4) [31]. Additionally, higher maternal vitamin B_12_ blood concentrations were associated with higher childhood eGFR_cystC_ (*p* < 0.01) (Table 4) [31]. Maternal folate and vitamin B_12_ blood concentrations were not associated with risk of microalbuminuria at the of age six (Table 4) [31].

#### 3.4.3. Vitamin D

Results from a prospective cohort study by Miliku et al. in the Netherlands among 4212 mothers and their children indicated that at age 6 years, children born to mothers who were vitamin D deficient (tested via blood samples) during mid-pregnancy had larger combined kidney volumes (*p* < 0.05) (Table 4) [30]. Lower maternal vitamin D levels during mid-pregnancy were associated with a higher eGFR_creat_ but not eGFR_cystC_ in school-age children [30]. Maternal vitamin D levels were positively associated with childhood blood creatinine levels (*p* < 0.05) but not blood cystatin C levels and risk of microalbuminuria at this age (Table 4) [30].

#### 3.4.4. Iron

One study in rural Bangladesh examined the association between prenatal food and multiple micronutrient supplementation and childhood kidney measures among 3267 mothers and their children at age 4–5 years [24]. This ‘Minimat’ trial by Hawkesworth et al. combined protein-energy and multiple micronutrient (MMN) inter­ventions and found that the estimated GFR, calculated from plasma cystatin C, was significantly higher (*p* = 0.04) in offspring whose mothers received 60mg vs. 30mg iron during pregnancy (Table 3) [24]. Maternal multiple micronutrient supplementation with iron and folate had no effect on childhood kidney volume and blood pressure (Table 3) [24].

#### 3.4.5. Famine

Two retrospective studies investigated the impact of exposure to famine in utero on offspring kidney function (Table 4) [27,28]. Huang et al. reported that exposure to the Chinese famine of 1959–1961 during gestation was associated with higher risk of proteinuria in offspring at approximately 30 years of age [27]. However, due to the long and imprecise duration of the Chinese famine, it is difficult to examine the effect of famine exposure at different stages of gestation [27]. Similarly, a follow-up study of offspring exposed to famine in utero during the Dutch winter famine of 1944–1945 (daily rations declined to under 1000 kcal/day) by Painter et al. indicated that the prevalence of microalbuminuria was significantly higher in adults (mean age of 50 years) exposed to famine in utero during mid-gestation compared to those born before or conceived after the famine [28]. Microalbuminuria in offspring exposed only in early or late gestation was not significantly different to those born before or conceived after the famine [28].

#### 3.4.6. Protein

In another prospective study among 3650 mothers and their six year-old children, Miliku et al. found that a higher maternal intake of total and vegetable protein, but not animal protein, during the first trimester of pregnancy was associated with higher eGFR_creat_, but not with kidney size, eGFR_cystC_, or microalbuminuria in school-aged children (Table 4) [29].

## 4. Discussion

This review of human studies highlights that maternal nutrition before conception and throughout gestation is a crucial factor that can influence the risk of renal dysfunction in offspring. Kidney disease is a major health issue, contributing to the global burden of disease and premature mortality. In 1990, chronic kidney disease was ranked as the 27th highest cause of mortality, but in the 2010 Global Burden of Disease study, it had risen to 18th indicating an increasing global burden of disease [34]. To our knowledge, this is the first review to systematically examine the evidence in relation to the effects of maternal nutrition during pregnancy on offspring kidney structure and function in humans. Nephron number is finalised at birth in humans [35] and is correlated with birth weight [36,37], emphasising the importance of the intrauterine environment for optimal fetal kidney development and function [38].

Although the influence of maternal nutrition during pregnancy on fetal renal development has been clearly demonstrated in numerous animal models, including large (e.g., sheep and pig) and small (e.g., mouse, rat and guinea pig) animals [39], there is relatively little information reported for humans in regard to the role of maternal nutrition in programming of renal disease in the offspring, with only 10 human studies identified in this review.

### 4.1. Maternal Nutrition and Offspring Kidney Structure or Function

#### 4.1.1. Maternal Micronutrient Deficiencies

Micronutrients are crucial in the enzymatic pathways of cell synthesis in the developing fetus, and therefore there is a high demand for them during pregnancy. Studies using rat models have shown that nephron number in offspring are reduced in proportion to maternal vitamin A levels [40] and Lelievre-Pegorier et al. report a 20% decrease in nephron number in adult rats born to mothers fed a vitamin A deficient diet [9]. This reduction of nephron number is most likely mediated through the active metabolite of vitamin A, retinoic acid, which is a key ligand for the cRET receptor that regulates uretic bud branching and vascular growth in the developing kidney [5,40].

Vitamin A deficiency is highly prevalent among pregnant women in some developing countries [41]. Three human studies that focused on maternal vitamin A levels during pregnancy were conducted in regions where there was high probability of in utero vitamin A deficiency (Nepal [26], India [32] and Egypt [33]). A similar finding was reported among Indian and Egyptian newborns by Goodyer et al. [32] and El-Khashab et al. [33], respectively where low maternal serum retinol concentration (vitamin A deficiency) was associated with fetal renal hypoplasia at birth, probably reflecting lower nephron numbers. One limitation in the study by Goodyer et al. [32] is that the offspring from India and Canada are unlikely to be comparable due to the variability in the confounding factors (i.e., maternal socioeconomic status, dietary patterns, pathogen exposure) between the two groups. A high percentage of missing data for some of the biochemical indices reported in the study by Stewart et al. may have reduced the power to detect differences in offspring blood pressure or microalbuminuria at 10 years of age between groups [26]. The varied kidney outcome measures, the different methods of assessing maternal vitamin A levels (supplementation vs venous blood) and limited number of studies meant that results across studies could not be meta-analysed. In addition, as these studies were conducted in three different vitamin A deficient rural populations, results cannot be generalised to other populations. The lack of intervention studies in humans is likely due to the potential teratogenic actions of retinoic acid at high doses [42], therefore vitamin A should not be supplemented indiscriminately. This further highlights the need for extended follow-up studies to determine the effect of vitamin A supplementation during pregnancy on offspring renal health.

Similar to vitamin A, gestational iron restriction in rats is associated with lower nephron number and hypertension in adult offspring [10]. Maternal iron supplementation during late nephrogenesis restores renal growth and nephron development in offspring of iron-deficient rats [10], highlighting the importance of optimal maternal iron levels during pregnancy, not only for normal renal development in utero, but also for the prevention of hypertensive disease in later life. Only one study was conducted in humans, which found that maternal iron supplementation did not have a major effect on offspring blood pressure at 4.5 years, although it was associated with elevated GFR [24], however, the follow-up period may be too short to observe differences in renal or cardiovascular outcomes. Iron deficiency anaemia is the most prevalent micronutrient deficiency globally [43]. Approximately two thirds of the world’s population experience iron deficiency, with pregnant women in both developed and developing countries at particularly high risk [43]. It is therefore important that additional intervention studies and longer follow-up periods in cohort studies are conducted in the future in order to assess the long-term effect of maternal iron supplementation on offspring renal health.

Folate is an essential B-vitamin, and together with vitamin B_12_, both are important for cell growth and are key methyl donors. They work closely together in homocysteine metabolism, generating methionine via methionine synthase [44]. Folate and vitamin B_12_ contribute to lowering homocysteine concentrations [45]. Although folic acid does not directly affect kidney development in experimental animals, given its effect on gene methylation, folate deficiency could impact nephrogenesis through epigenetic modulation [46]. Animal studies indicate that elevated homocysteine levels may result in glomerular damage, and folic acid supplementation lowers plasma creatinine concentration and urinary albumin excretion induced by hyper-homocysteinemia [47]. Not many studies have explored the effect of maternal folate concentrations or folic acid supplementation during pregnancy on human offspring kidney structure or function. Among 6–8 year-old children in rural Nepal, Stewart et al. found that maternal folic acid supplementation was associated with a reduction in microalbuminuria [25]. However, Miliku et al. did not observe an association between maternal folic acid supplementation or maternal folate concentrations in early pregnancy and risk of microalbuminuria in six year-old children [31]. The differences in results may be explained by the different study populations with varied confounding factors, such as maternal socioeconomic status and dietary habits. Results from the study by Miliku et al. suggested that maternal folate concentration in early pregnancy had a positive correlation with childhood combined kidney volume, but the study by Hawkesworth et al. indicated no effect of early maternal multiple micronutrient supplementation or food supplementation with iron and folate on offspring’s kidney volume. Compared to self-reported folic acid intake or supplementation interventions, plasma folate concentration is more accurate in measuring maternal folate status. This could explain study differences in maternal folate concentrations being associated with childhood kidney volume, rather than folic acid supplementation. Given that both observational and animal experimental studies have shown associations between elevated homocysteine concentrations and an accelerated decline in renal function [47,48], maternal folate supplementation during pregnancy could potentially improve renal function in offspring through its ability to reduce homocysteine. However, further studies are needed to confirm this.

Experimental studies in animals have shown that maternal vitamin D deficiency during pregnancy may affect offspring kidney health. Rat offspring born to mothers who were fed a vitamin D deficient diet during pregnancy and lactation had a 20% increase in nephron number compared with the controls [49]. Another experimental study in mice found a higher number of glomeruli and a delayed maturity of the glomeruli in kidneys of vitamin D deplete offspring [50]. Not many studies have examined the association of maternal vitamin D levels during pregnancy and childhood kidney structure and function, and this review only found one study in humans [30]. However, in line with the observations from experimental studies, Miliku et al. observed that maternal vitamin D levels during pregnancy may influence childhood kidney structure and function. Lower maternal vitamin D levels in blood were associated with a higher eGFR and larger combined kidney volume in school-age offspring in this study. However, the impact of maternal vitamin D deficiency on offspring kidney development needs further exploration. Further studies are needed to examine the underlying mechanism and to identify the long-term renal consequences of maternal vitamin D deficiency. Given the high prevalence of vitamin D deficiency among adult women of childbearing age in many parts of the world [51,52,53,54,55,56] and that pregnant women are at higher risk, it is imperative that we gain an understanding of how maternal vitamin D deficiency can potentially affect kidney development in offspring.

#### 4.1.2. Maternal Energy and Protein Restriction

Numerous maternal undernutrition models have been established in animal research that alter the maternal diet to try and mimic the human condition. Animal models of maternal protein or energy restriction during gestation have been consistently associated with lower nephron numbers, elevated blood pressure and renal dysfunction in offspring [6,7,57,58,59,60,61]. In these studies, the kilojoule restriction varied from 30% to 70% of normal intake. The level of protein restriction varied widely, contributing from 5% to 12% of total energy intake, with control levels being set at 18–20% of total energy intake. However, there are obvious ethical reasons why such intervention studies cannot be conducted in humans. To examine the link between maternal protein and energy malnutrition during pregnancy and offspring kidney health, there is a need for prospective cohort studies that examine specific populations likely to have been exposed to maternal malnutrition in pregnancy.

This systematic review found that there were only two studies that have investigated this link. Both the Dutch Hunger Winter of 1944–1945 [28] and the Chinese famine of 1959–1961 [27] provided an opportunity to retrospectively analyse the impact of maternal energy restriction on offspring renal function. However, epidemiological studies investigating the relationship between intrauterine environment exposure of the fetus and kidney outcomes 70 years later are subjected to numerous limitations. It is unlikely that confounding factors during maternal pregnancy (such as maternal health status, smoking and weight status) were adequately adjusted for. Furthermore, the accuracy and reliability of reported maternal nutritional status is poor and the inability to account for any black-market supplies during the famine. Results therefore have to be interpreted with caution.

Maternal protein restriction during pregnancy is the most widely studied experimental animal model of in utero nutritional programming. Extensive evidence from animal studies indicates that restricted maternal protein intake in pregnancy is linked to lower birth weight, impaired nephron endowment, elevated blood pressure and reduced GFR [57,58,60]. Only one human study was identified in the current review that investigated the relationship between maternal protein intake during pregnancy and offspring renal health [29]. That study suggests that a higher first-trimester maternal total protein and vegetable protein intake was associated with a higher eGFR in offspring at 6 years [29]. Younger mothers in lower socioeconomic groups within developed countries are more likely to have lower dairy and meat protein intakes in relation to carbohydrate [62]. This may lead to a higher risk of the associated renal impairment in their offspring. Given the vast evidence from animal studies, it is recommended that further prospective cohort studies in populations where there is a high prevalence of maternal protein deficiency be conducted to further investigate the associated risk of kidney diseases in offspring during adulthood.

### 4.2. Quality of Included Studies

The overall quality of the included studies was moderate. Overall, seven [24,25,26,28,29,30,31] out of the 10 studies met the criteria for a positive quality rating based on study generalizability, bias and validity. Three studies [27,32,33] were rated neutral, indicating that the overall generalizability of these study outcomes should be interpreted with caution when applied to a general population. The included studies were primarily observational (cohort studies), providing a low level of evidence to which causality cannot be applied. Limited studies on the different aspects of maternal nutritional status and measurement of offspring kidney outcomes were diverse across studies, thus weakening the strength of the evidence. There was also a lack of consistency in the follow-up time-point when offspring kidney structure and/or function were assessed, thus limiting comparability between studies. All of the positive-rated studies used different models of statistical analysis taking into account the various confounders. To improve study quality and reporting, we suggest future research include these aspects of study design.

### 4.3. Strengths and Limitations

Strengths of this systematic review include: (1) a comprehensive search strategy across five databases with no date restrictions, (2) detailed data extraction allowing comparison between studies, (3) methodological quality in line with the PRISMA statement, and (4) development of, and adherence to an evidence-based protocol registered with PROSPERO. Limitations of this review include: (1) failure to include studies published in languages other than English, and (2) meta-analysis could not be performed due to the heterogeneity between studies in kidney outcome measures, the large variation in the age at follow-up of offspring, and the diverse components of maternal diets that were assessed.

### 4.4. Implication for Practice

A growing body of evidence in animal models on nutritional programming indicates that nutrition-related factors in pregnancy could permanently alter or program offspring nephrogenesis, however parallel evidence is lacking in humans. The available evidence in humans suggests that fetal undernutrition predisposes individuals to the development of renal disease, as it does in animals. Alterations in kidney structure and/or function have been shown in offspring whose mothers took part in a randomized controlled trial of MMN supplements before and during pregnancy and in adult offspring of pregnant women exposed to the Dutch winter famine. This emphasised the importance of close monitoring of renal function in children exposed to in utero undernutrition, especially in populations with high risk of undernutrition, for example in some populations in India and China, where for generations the quality of their diet has been poor [61].

### 4.5. Implication for Research

Overall, while the number of human studies investigating the DOHaD hypothesis from a kidney health perspective are relatively small, the majority of the studies included in this review have been published since the year 2010, indicating that this is an emerging area of interest. Understanding the role of developmental programming and an adverse intrauterine environment will assist in developing preventive strategies that aim to alleviate the progression of chronic diseases, including renal disease. There are a number of key issues that should be addressed in future research investigating the relationship between maternal nutrition during pregnancy and offspring kidney structure and function. These are:

1. Prospective studies should be designed to ensure accurate assessment of maternal nutritional status and dietary intake at specific times in gestation;
2. High quality longitudinal studies with longer follow-ups beyond childhood are needed;
3. Consideration of the use of common outcomes for maternal and offspring kidney health to facilitate meta-analysis of results across studies.

## 5. Conclusions

This study has systematically reviewed the evidence on the relationship between maternal nutrition during pregnancy and offspring kidney structure and function in humans. This review indicates that emerging evidence from randomized controlled maternal micronutrient intervention trials suggest some protective effects of antenatal vitamin A, iron and folate supplementation on fetal kidney health in humans. However, further high-quality intervention and longitudinal studies with follow-up periods beyond childhood are warranted to better characterise relationships with offspring renal function. With increasing knowledge regarding the vital contribution of optimal maternal health, dietary intake and nutritional status during pregnancy in developmental programming and their transgenerational effects [2], identifying at-risk populations and implementing locally adapted public health interventions prior to and during pregnancy are key to developing future preventative strategies. This has the potential to reduce the long-term disease risk for the individual and for subsequent generations, and also has substantial long-term economic benefits on health care costs worldwide.

## Figures and Tables

**Table 1:** Description of intervention studies.

References	Setting	Aims, Design	Sample size	Details of Intervention	Intervention Length	Offspring Age at Follow-Up (Years), Sex
Intervention Studies (*n* = 3)						
Stewart et al. 2010 [26]	Rural, low-lying Sarlahi District of Nepal	**Aim**: To determine the effect of maternal Vitamin A or β-Carotene supplementation from pre-pregnancy through the post-partum period on childhood cardiovascular risk factors **Design**: Cluster-Randomized, double blind, placebo-controlled trial of vitamin A or β-Carotene supplementation.	Placebo—4270 Vitamin A—4580 β-Carotene—4268	Women were randomized by ward to receive weekly supplementation with: 1. Placebo 2. 7000 mg retinol equivalents of preformed vitamin A (23,300 IU retinyl palmitate) 3. 7000 mg retinol equivalents of β-Carotene (42 mg)	Before, during and after pregnancy	Mean ± SD: 10.4 ± 0.71 Males (Placebo: 51.4%, Vitamin A: 50.9%, β-Carotene: 51.1%) Females (Placebo: 48.6%, Vitamin A: 49.1%, β-Carotene: 48.9%)
Stewart et al. 2009 [25]	Rural, low-lying Sarlahi District of Nepal	**Aim**: To examine the impact of antenatal micronutrient supplementation on cardio-metabolic risk in the offspring at 6–8 years. **Design**: Community-based, cluster randomized, controlled trial of antenatal micronutrient supplementation.	Control—735 Folic acid—658 Folic acid + iron—674 Folic acid + iron + zinc—708 MMS—749	Pregnant women were provided with daily supplements: 1. Vitamin A alone (Control) 2. Folic acid (400 mg) 3. Folic acid + iron (60mg) 4. Folic acid + iron + zinc (30 mg) 5. MMS containing folic acid, iron, zinc, and an additional 11 vitamins and minerals All supplements contained 1 mg retinol equivalents of vitamin A in the form of retinyl acetate.	From the time of enrolment (early pregnancy) through 3 months postpartum	Mean: 7.5 Numbers for each sex not specified.
Hawkesworth et al. 2013 [24]	International Centre for Diarrheal Disease Research, Bangladesh.	**Aim**: To assess the association between prenatal food and micronutrient supplementation and childhood blood pressure and kidney function **Design**: Follow up study of infants at 4.5 years from mothers who participated in the Maternal and Infant Nutrition Interventions, MatLab (MINIMat) Randomised Control Trial.	Early food + Fe30F—427 Early food + Fe60F—416 Early food + MMS—431 Late food + Fe30F—413 Late food + Fe60F—437 Late food + MMS—402	2 separate nutritional interventions in pregnancy: 1. Food supplementation Food supplements (608kcal/day energy and 18 g/day of vegetable protein) were provided either: (a) early in pregnancy (~ 9 week gestation; early food) or (b) late in pregnancy (~ 20 week gestation; late food). 2. Micronutrient supplementation (a) 30mg of iron and 400μg of folate (Fe30F)/day (b) 60mg of iron and 400μg of folate (Fe60F)/day (c) 15 micronutrients ≥ the RDA, including 30mg iron and 400μg folate (MMS)/day	Early Food: 21 weeks (around 9 week of gestation to week 30 gestation) Late Food: 10 weeks (around 20 week of gestation to week 30 gestation)	Mean ± SD: 4.6 ± 0.1 Male (50.5%) Female (49.5%)

MMS: Multiple Micronutrient Supplementation; SD: standard deviation.

**Table 2:** Description of observational studies.

References	Setting	Aims, Design	Inclusion/Exclusion Criteria	Study Population	Offspring Age at Follow-Up (Years), Sex
Observational Studies (*n* = 7)					
Goodyer et al. 2007 [32]	St. John’s Medical College Hospital, Bangalore, India and Royal Victoria Hospital, Montreal, Canada	**Aim:** To determine the prevalence of maternal vitamin A deficiency and its relationship to offspring nephron endowment from normal pregnant women from Bangalore (India) and Montreal (Canada) **Design:** Prospective cohort.	**Inclusion:** Healthy women with uncomplicated pregnancies **Exclusion:** Mothers on vitamin A supplements, high risk and medically complicated pregnancies, twin, diabetic and growth restricted pregnancies, significant fetal structural or genetic abnormalities, fetuses with significant pyelectasis or renal anomalies and pregnancies delivered <32 weeks of gestation.	Montreal: 48 Bangalore: 46	2–6 weeks of age Numbers for each sex not specified.
El-Khashab et al. 2013 [33]	Gynecology and Obstetrics Hospital, Ain Shams University, Cairo, Egypt	**Aim:** To assess the Vitamin A status of a cohort of Egyptian pregnant women and their newborns and to determine the potential effect of maternal Vitamin A deficiency during pregnancy on the neonatal kidney size. **Design:** Cross-sectional.	**Inclusion**: Healthy mothers, aged 19–39 years, with singleton uncomplicated pregnancy. Full term, healthy offspring with no morbidities. **Exclusion**: 1. Mothers with a history of exposure to teratogens, high-risk and complicated pregnancies, multiple gestations, antenatal diagnosis of intrauterine growth restriction, renal anomalies, fetal structural abnormalities, on vitamin A supplementation. 2. Offspring born <37 weeks, birth weight <2.5 kg, birth asphyxia, congenital anomalies or dysmorphic features.	Vitamin A deficiency: serum retinol concentration ≤0.7 umol/L (*n* = 16) Vitamin A sufficient: serum retinol concentration >0.7 umol/L (*n* = 64)	≤3 days Numbers for each sex not specified.
Miliku, K. et al. 2017 [31]	Generation R study: Population-based prospective cohort study from fetal life onward in Rotterdam, Netherlands	**Aim:** To examine the associations of folate, vitamin B_12_, and homocysteine concentrations during first trimester of pregnancy and at birth with kidney outcomes in school-aged children **Design:** Prospective cohort study.	**Inclusion:** Singleton live-born children from mothers with nutritional data and with at least one kidney measurement.	4226 mothers-child pairs Maternal venous blood samples were collected in early-pregnancy (median gestational age 13.2 weeks, range 12.2–14.8 weeks). Deficient folate <7 nmol/L Normal folate ≥7 nmol/L	Median (95% range): 6.0 (5.9–6.3) Deficient folate Male (51.3%) Female (48.7%) Normal folate Male (49.9%) Female (50.1%)
Miliku, K. et al. 2016 [30]	Generation R study: Population-based prospective cohort study from fetal life onward in Rotterdam, Netherlands	**Aim:** To examine the associations of circulating vitamin 25-hydroxyvitamin D (25(OH)D) levels during mid-pregnancy and in cord blood at birth with childhood kidney outcomes **Design:** Prospective cohort study.	**Inclusion:** Singletons with available information on maternal vitamin D blood levels during mid-pregnancy and have measurements on kidney ultrasound, creatinine and cystatin C from blood, and albumin and creatinine from urine samples at the age of 6 years. **Exclusion:** Children with evidence of congenital kidney abnormalities on ultrasound examination or with abnormally high urinary albumin–creatinine ratio	4212 mothers-child pairs Maternal venous blood samples were collected in mid-pregnancy (median gestational age 20.3 weeks, range 18.5–23.3 weeks). Total 25(OH)D was reported as the sum of 25(OH)D_2_ and 25(OH)D_3_ measured in plasma. Maternal vitamin D status: severely deficient <25.0 nmol/L deficient 25–49.9 nmol/L Sufficient 50.0–74.9 nmol/L Optimal ⩾75.0 nmol/L	Median (95% range): 6.0 (5.6–7.4) Male (49.6%) Female (50.4%)
Huang et al. 2014 [27]	Chinese famine of 1959–1961	**Aim:** To describe the long term effects of exposure to the Chinese famine of 1959–1961 during gestation and early postnatal life on the levels of proteinuria in adulthood **Design:** Retrospective cohort.	**Inclusion:** Restricted study to women born from 1957 to 1965 and living in Hebei, Zhejiang and Jiangsu provinces. **Exclusion:** 18.3% of rural sample, 12.9% of urban sample due to missing data on outcome variable. Famine (<1500 calories daily per capita)	Rural: *n* = 51,978 (i) Pre-famine (1957–1958): *n* = 2050 (ii) Famine (1959–1961): *n* = 6396 (iii) Post-famine (1962–1963): *n* = 24,739 (iv) Unexposed group (1964–1965): *n* = 18,793 Urban: *n* = 4563 (i) Pre-famine (1957–1958): *n* = 241 (ii) Famine (1959–1961): *n* = 518 (iii) Post-famine (1962–1963): *n* = 1594 (iv) Unexposed group (1964–1965): *n* = 2210	Approx. 30 All female only
Painter et al. 2005 [28]	The Dutch Famine Birth Cohort study, the Netherlands	**Aim:** To describe the long-term effects of prenatal exposure to famine on microalbuminuria and renal function **Design:** Retrospective cohort.	**Inclusion:** Exposed: Infants born in 1945 and were exposed to famine in utero. Unexposed: Infants born before or conceived after the famine. Famine <1000 calories daily	288 (40%) of the 724 participants studied had been exposed to famine in utero. Exposed (*n* = 288): 3 groups; 16-week periods Early gestation: *n* = 65 Mid gestation: *n* = 104 Late gestation: *n* = 119 Unexposed (*n* = 506): Born before: *n* = 207 Conceived after: *n* = 229	Mean (95% range): 50 (48–53) Male Early: 46% Mid: 42% Late: 47% Female Early: 54% Mid: 58% Late: 53%
Miliku, K. et al. 2015 [29]	Generation R study: Population-based prospective cohort study from fetal life onward in Rotterdam, Netherlands	**Aim:** To assess the associations between first-trimester maternal total, animal and vegetable protein intake during pregnancy and offspring kidney outcomes at 6 years **Design:** Prospective cohort study.	**Inclusion:** 1. Living in the study area at delivery 2. Delivery between April 2002 and January 2006 **Exclusion:** 1. Twin pregnancies 2. Loss to follow-up 3. Withdrawal of consent	3650 mother-child pairs (78%) of the 4658 children with maternal nutritional data available and who attended follow-up at age 6 with successful kidney outcome measures	Median (95% range): 6.1 (5.6–7.3) Male (49.9%) Female (50.1%)

**Table 3:** Results of intervention studies.

References	Offspring Kidney-Related Outcomes Reported			Conclusion, Limitations & Recommendations
	Measures	Significant difference between Groups	Results	
Intervention Studies (*n* = 3)				
Stewart et al. 2010 [26]	BP (mmHg)	No significant difference between vitamin A or β-Carotene groups and placebo group (in crude model or after adjustment for child’s age or gender)	Adjusted difference (Mean (S.D.)) Systolic BP Vitamin A: −0.19 (−0.94, 0.56) β-Carotene: −0.21 (−1.00, 0.58) Diastolic BP Vitamin A: −0.03 (−0.90, 0.83) β-Carotene: −0.19 (−1.07, 0.70)	**Limitations** 1. High percentage of missing data for some biochemical measures reduced power to detect differences between groups. 2. The prevalence of hypertension and micro-albuminuria was only 5%, reflecting a low disease risk and power to detect risk factors. 3. Observations only applicable to rural population **Strengths** 1. Presents data from a large cohort of more than 13,000 children 2. More than half of all women consumed >80% of their intended supplements 3. A high rate of follow-up was achieved, with no differential losses and a high degree of comparability across groups. 4. Study design minimized the risk of confounders **Conclusion** No evidence of an overall effect of maternal supplementation with vitamin A or β-Carotene on BP or risk of microalbuminuria.
	Risk of hypertension	No overall difference between supplement groups	Adjusted OR (95%) Vitamin A: 1.14 (0.72, 1.79) β-Carotene: 0.81 (0.46, 1.44)	
	Risk of microalbuminuria (≥30 microalbumin/creatinine mg/g)	No overall difference between supplement groups	Adjusted OR (95%) Vitamin A: (0.40, 3.71) β-Carotene: 0.68 (0.20, 2.30)	
Stewart et al. 2009 [25]	BP (mmHg)	No difference between intervention groups and control for both systolic and diastolic BP	-	**Limitations** High numbers of missing data, decreased the sample size and power to find significant differences among treatment groups. **Strengths** This study provides unique data for the effects of micronutrient supplementation during pregnancy on risk factors for chronic disease from a RCT with high compliance and a high rate of follow-up. **Conclusion** 1. No impact of antenatal MMS on BP. 2. 36% reduction in the risk of microalbuminuria when all supplementation groups containing folic acid were compared with the control, suggesting a protective role of folic acid on kidney function.
	Risk of microalbuminuria (≥3.4 albumin/creatinine mg/mmol)	Significant reduction among mothers in the folic acid and folic acid + iron + zinc groups compared with the control	OR (95% CI) Folic acid:0.56 (0.33, 0.93) Folic acid + iron: 0.77 (0.49, 1.22) Folic acid + iron + zinc: 0.53 (0.32, 0.89) Multiple micronutrient: 0.70 (0.44, 1.11)	
Hawkesworth et al. 2013 [24]	Diastolic BP (mmHg)	Lower in early food than late food (*p* = 0.01)	Mean difference of 0.74 (95% CI: 0.18, 1.30)	**Limitations** 1. Women only randomly assigned to encouragement to food supplementation rather than provided with food supplementation itself. 2. The study lacked control arms and a MMS arm that also contained iron. 3. Age of offspring may be too young to see differences in renal or cardiovascular outcomes. **Strengths** Large sample size, good retention of participants **Conclusion** Overall there was limited evidence for long-lasting impacts of pregnancy supplementation on offspring blood pressure or markers of kidney function.
		Higher in MMS compared with iron and folate (*p* = 0.03)	Mean difference of 0.65 (95% CI: 0.06, 1.24)	
		No difference between high and low iron intervention	-	
	Systolic BP (mmHg)	No effect of food or nutritional supplementation	-	
	Kidney Volume (cm^3^/m^2^)	No effect of food or nutritional supplementation	-	
	eGFR from plasma Cystatin C (ml/(min × 1.73 m^2^)	No difference between early or late food	-	
		Higher in offspring whose mothers received 60mg of iron during pregnancy vs. 30mg (*p* = 0.04)	Mean difference of 4.98 (95% CI: 0.30, 9.67)	

MMS: Multiple Micronutrient Supplementation; BP: Blood Pressure; eGFR: estimated glomerular filtration rate; CKD: Chronic kidney disease; RCT: Randomized controlled trial.

**Table 4:** Results of observational studies.

References	Offspring Kidney-Related Outcome Reported			Conclusion, Limitations & Recommendations
	Measures	Significance of Results	Results	
Observational Studies (*n* = 7)				
Goodyer et al. 2007 [32]	Mean combined renal volume (ml) at 1 month	Significantly smaller in Bangalore (VAD) than that in Montreal (VAS) (*p* < 0.001)	22.3 ± 7.0 vs. 40.1 ± 11.0	**Conclusion** Vitamin A deficiency has an additional effect on nephron number as reflected in kidney volume. **Recommendations** 1. Future study should target the assessment of renal volume during the immediate newborn period prior to discharge from hospital. 2. Cord cystatin C measurements might be used to estimate fetal renal function mass.
	Mean combined renal volume/Body surface area (ml/m^2^)	Significantly lower in Bangalore (VAD) than that in Montreal (VAS) (*p* < 0.01)	113.7 ± 33.3 vs. 184.2 ± 43.6	
	Mean maternal retinol + renal size at 1 month in Bangalore newborns	No significant correlation (*p* > 0.4)	*r* = 0.159 (*n* = 25 only)	
El-Khashab et al. 2013 [33]	Mean longitudinal axis (cm)	Significantly lower in both kidneys of newborns delivered to VAD mothers compared with the newborns delivered to VAS mothers (Right: *p* = 0.005; Left: *p* = 0.008)	**Right Kidney** VAD: 3.12 ± 0.96 VAS: 3.86 ± 0.51 **Left Kidney** VAD: 3.25 ± 1.11 VAS: 3.92 ± 0.41	**Conclusion** Maternal VAD during pregnancy may decrease the renal size in the infant at birth. **Limitation** 1. Did not include the follow-up of renal size and the effect on renal functions of the infants 2. Small sample size **Recommendations** Longitudinal studies on larger scale needed to validate the results and to explore the effect on renal functions
	Mean transverse axis (cm)	Significantly lower in both kidneys of newborns delivered to VAD mothers compared with the newborns delivered to VAS mothers (Right: *p* = 0.008; Left: *p* = 0.05)	**Right Kidney** VAD: 1.57 ± 0.44 VAS: 1.94 ± 0.31 **Left Kidney** VAD: 1.63 ± 0.44 VAS: 1.92 ± 0.33	
	Mean renal volume (cm^3^)	Significantly lower in both kidneys of newborns delivered to VAD mothers compared with the newborns delivered to VAS mothers (Right: *p* = 0.002; Left: *p* = 0.002)	**Right Kidney** VAD: 7.42 ± 2.45 VAS: 9.5 ± 2.31 **Left Kidney** VAD: 7.95 ± 1.36 VAS: 9.96 ± 2.41	
	Combined renal volume (cm^3^)	Significantly lower in newborns delivered to VAD mothers compared with the newborns delivered to VAS mothers (*p* = 0.001)	VAD: 15.95 ± 2.47 VAS: 19.01 ± 3.49	
	Combined renal volume and maternal serum retinol concentrations	Significant positive correlation (*p* = 0.001)	*r* = 0.48	
Miliku, K. et al. 2017 [31]	Combined kidney volume (cm^3^)	Higher maternal folate concentration was associated with a larger childhood combined kidney volume. (*p* < 0.01)	Difference: 1.16 (95% CI: 0.47, 1.85) per 1 SD in folate	**Limitations** Results apply to a relatively healthy sample of pregnant women and children, generalizability of results to other populations should be interpreted with caution. **Strengths** 1. The largest prospective population-based cohort study examining the associations of folate, vitamin B_12_ and homocysteine concentrations during fetal life with childhood kidney outcomes. 2. Detailed measurements on kidney outcomes were obtained. **Conclusion** 1. Maternal higher folate and lower homocysteine concentrations are associated with larger childhood combined kidney volume. 2. Maternal higher vitamin B_12_ and lower homocysteine concentrations were associated with higher childhood eGFR_cystC_. 3. No association between folic acid supplement intake and kidney outcomes. 4. Effect sizes presented are small. **Recommendations** These findings should be considered as hypothesis generating and require further replication. Additional follow-up studies are warranted to examine the long term consequences for the risk of kidney diseases in later life.
		Maternal vitamin B_12_ concentration was not associated with childhood combined kidney volume.	Difference: 0.47 (95% CI: −0.17, 1.11) per 1 SD in vitamin B_12_	
		Higher maternal homocysteine concentration was associated with a smaller childhood combined kidney volume. (*p* < 0.01)	Difference: −1.44 (95% CI: −2.09, −0.79) per 1 SD in homocysteine	
		No association between maternal folic acid supplement intake during pregnancy and childhood combined kidney volume.	-	
	eGFR_creat_ (ml/min per 1.73 m^2^)	Maternal folate, vitamin B_12_ and homocysteine concentration were not associated with childhood eGFR_creat_.	**Folate** Difference: 0.01 (95% CI: −0.65, 0.67) per 1 SD in folate **Vitamin B_12_** Difference: 0.20 (95% CI: −0.43, 0.83) per 1 SD in vitamin B_12_ **Homocysteine** Difference: −0.55 (95% CI: −1.15, 0.05) per 1 SD in homocysteine	
		No association between maternal folic acid supplements intake during pregnancy and childhood eGFR_creat_.	-	
	eGFR_cystC_ (ml/min per 1.73 m^2^)	Maternal folate concentration was not associated with childhood eGFR_cystC_.	Difference: −0.03 (95% CI: −0.63, 0.58) per 1 SD in folate	
		Higher maternal vitamin B_12_ concentration was associated with a higher childhood eGFR_cystC_. (*p* < 0.01)	Difference: 1.00 (95% CI: 0.43, 1.57) per 1 SD in vitamin B_12_	
		Higher maternal homocysteine concentration was associated with a lower childhood eGFR_cystC_. (*p* < 0.05)	Difference: −0.57 (95% CI: −1.13, −0.02) per 1 SD in homocysteine	
		No association between maternal folic acid supplement intake during pregnancy and childhood eGFR_cystC_.	-	
	Microalbuminuria	Maternal folate, vitamin B_12_ and homocysteine concentration were not associated with risk of microalbuminuria.	**Folate** OR 0.97 (95% CI: 0.85, 1.10) per 1 SD in folate **Vitamin B_12_** OR 1.06 (95% CI: 0.95, 1.19) per 1 SD in vitamin B_12_ **Homocysteine** OR 1.08 (95% CI: 0.98, 1.20) per 1 SD in homocysteine	
		No association between maternal folic acid supplement intake during pregnancy and risk of microalbuminuria.	-	
Miliku, K. et al. 2016 [30]	Combined kidney volume (cm^3^)	Larger in children of mothers who were vitamin D deficient during pregnancy compared with children of mothers who had optimal 25(OH)D levels. (*p* < 0.05)	Difference: 1.92 (95% CI: 0.11, 3.74)	**Limitations** 1. Mothers of the children who were lost to follow-up had on average lower 25(OH)D levels and were on average lower educated, suggesting that the study population had a bias toward a more healthy population. 2. Same cut-offs of 25(OH)D were used for pregnant woman as the documented levels for general population 3. Childhood dietary data at the age of 6 years were not available. 4. Microalbuminuria was evaluated using urine albumin–creatinine ratio from a random urine sample, instead of first-morning void samples. 5. Residual confounding by other lifestyle factors might be present. **Strengths** 1. Prospective design from fetal life onward within a large population-based cohort. 2. 25(OH)D levels were used, which are the best and the most widely used indicator of vitamin D status. 3. Well-established methods were used to measure kidney size and function. **Conclusion** 1. Mothers who were 25(OH)D deficient had children with larger combined kidney volumes. 2. Lower maternal 25(OH)D levels were associated with an increased eGFR_creat_ in school-age children. **Recommendations** Further studies are needed to replicate the observations, examine the underlying mechanisms and to identify the long-term clinical consequences.
	eGFR_creat_ (ml/min per 1.73 m^2^)	Maternal 25(OH)D levels were inversely associated with lower childhood eGFR_creat_. (*p* = 0.02)	Difference: −0.94 (95% CI: −1.73, −0.15) per 1 SD in 25(OH)D	
	eGFR_cystC_ (ml/min per 1.73 m^2^)	Maternal 25(OH)D levels were not associated with eGFR_cystC_.	Difference: − 0.29 (95% CI: −0.99, 0.41) per 1 SD in 25(OH)D	
	Microalbuminuria	Maternal 25(OH)D levels were not associated with risk of microalbuminuria.	OR: 0.93 (95% CI: 0.80, 1.09) per 1 SD in 25(OH)D	
	Creatinine levels from blood (μmol/L)	Maternal 25(OH)D levels were associated with higher childhood creatinine levels. (*p* < 0.05)	0.32 (95% CI: 0.07, 0.58) per 1 SD in 25(OH)D	
	Cystatin C levels from blood (μg/L)	Maternal 25(OH)D levels were not associated with childhood cystatin C levels.	2.32 (95% CI: −1.55, 6.19) per 1 SD in 25(OH)D	
Huang et al. 2014 [27]	Proteinuria	**Rural:** Famine exposure was associated with 54% higher odds of having a higher concentration of protein in the urine compared with the unexposed group (*p* = 0.029)	OR: 1.54 (95% CI: 1.04, 2.28)	**Limitations** 1. Dipstick test of urine specimens is less sensitive and accurate than other methods. 2. Assumption that women were living in the same country as when they were born. 3. Famine cohort may represent a more selective and robust population compared with post-famine cohorts. May result in underestimation of the effects of famine exposure on adult health outcomes. 4. The long and imprecise duration of the Chinese famine of 1959–61 did not permit isolation of prenatal and postnatal exposures. **Strengths** First study to examine the effects of the Chinese famine of 1959–1961 on renal function. **Conclusion** Exposure to the Chinese famine of 1959–1961 during gestation was associated with higher level of proteinuria three decades later after the exposure in rural residents, but the effect size was small.
		**Rural:** No association between famine exposure and the concentration of protein in urine for the pre-famine group (*p* = 0.380)	OR: 1.29 (95% CI: 0.73, 2.26)	
		**Rural:** No significant difference in the levels of proteinuria between post-famine and unexposed group (*p* = 0.051)	OR: 1.26 (95% CI: 0.99, 1.59)	
		**Urban:** No significant difference in the occurrence of proteinuria in the pre-famine, famine and post-famine group when compared with the unexposed group	-	
Painter et al. 2005 [28]	Prevalence of Microalbuminuria	Significantly higher in those who were exposed to famine in mid gestation than in people who were not exposed prenatally (*p* = 0.05)	12% vs. 7%	**Limitations** 1. Conclusions are based on non-invasive outcome measures only. 2. Small number of individuals in each of the exposure groups limited the ability to make firm statements about the precise timing of the effects 3. Small sample size. **Conclusion** Exposure to famine in mid-gestation is linked to a 3.2-fold increase in occurrence of microalbuminuria in adulthood and a 10% decrease in creatinine clearance.
		Not significantly increased in early (*p* = 0.3) or late gestation (*p* = 0.8)	-	
	Mean creatinine clearance (mL/min)	Decreased in those exposed to famine in mid-gestation compared to those conceived after (*p* < 0.01)	Decrease of 11.9 (95% CI: 4.0, 19.8)	
		Those conceived after famine had the highest mean clearance	-	
		A gender- and age-adjusted decrease in those born before the famine compared to those conceived after (*p* < 0.01)	Decrease of 16.4 (95% CI: 7.4, 25.4)	
Miliku, K. et al. 2015 [29]	eGFR_creat_ (mL × min^−1^ × 1.73 m^−2^)	Higher with higher first trimester maternal total protein intake (*p* < 0.05)	Difference: 0.06/g total protein intake (95% CI: 0.01, 0.12)	**Limitations** FFQ validated only in Dutch elderly women (55–75 years old), not specifically in pregnancy. **Strengths** Prospective design from fetal life onward within a large population-based cohort **Conclusion** 1. A higher maternal intake of total and vegetable protein, but not animal protein, during the first trimester of pregnancy is associated with higher eGFRcreat but not with kidney size, eGFRcyst C, or microalbuminuria in school-aged children. 2. First-trimester maternal protein intake was positively associated with eGFRcreat in 6 year-old children. 3. The associations with eGFR were stronger for vegetable than for animal protein intake during pregnancy. 4. Child protein intake at the age of 1 year does not affect the association between maternal protein intake and childhood kidney health. **Recommendations** Follow-up studies are needed to explore whether protein intake in pregnancy affects the risk of kidney diseases in adulthood. Further studies are needed to investigate the underlying mechanisms.
		Strongly associated with first trimester maternal vegetable protein intake (*p* < 0.05)	Difference: 0.22/g vegetable protein intake (95% CI: 0.10, 0.35)	
		Not significantly associated with first trimester maternal animal protein intake	-	
	eGFR_cystC_ (mL × min^−1^ × 1.73 m^−2^)	Not associated with first trimester maternal total protein intake	-	
	Kidney volume (cm^3^)	Not associated with first trimester maternal total protein intake	-	
	Microalbuminuria	Not associated with first trimester maternal total protein intake	-	
	Serum creatinine (umol/L)	First trimester maternal total protein intake associated with lower concentrations of creatinine	−0.02/g total protein intake (95% CI: −0.04, −0.01)	
		First trimester maternal vegetable protein intake associated with lower concentrations of creatinine	−0.07/g vegetable protein intake (95% CI: −0.11, −0.03)	
	Serum cystatin C (ug/L)	Not associated with first trimester maternal total protein intake	-	
		Not associated with first trimester maternal vegetable protein intake	-	

## Supplementary Materials

The following are available online at http://www.mdpi.com/2072-6643/10/2/241/s1, Table S1: Results of study quality based on the American Dietetic Association (ADA) Quality Criteria.
