# Supplementary material for: The Relationship between Maternal Nutrition during Pregnancy and Offspring Kidney Structure and Function in Humans: A Systematic Review
Source: Nutrients. 2018 Feb 21;10(2):241. doi: 10.3390/nu10020241 (PMC5852817; doi:10.3390/nu10020241)
Supplement: Supplementary file 1 [file nutrients-10-00241-s001.pdf]

Table S1. Results of study quality based on the American Dietetic Association (ADA) Quality Criteria [a].

Author	Quality Rating [b]	Relevance Q1	Relevance Q2	Relevance Q3	Relevance Q4	Validity Q1	Validity Q2	Validity Q3	Validity Q4	Validity Q5	Validity Q6	Validity Q7	Validity Q8	Validity Q9	Validity Q10
Interventional Studies (*n* = 3)															
Stewart et al. 2010 [26]	+	No	Yes	Yes	Yes	Yes	Yes	Yes	Yes	Unclear	Yes	Yes	Yes	Yes	Yes
Stewart et al. 2009 [25]	+	Yes	Yes	Yes	Yes	Yes	Yes	Yes	Yes	Unclear	Yes	Yes	Yes	Yes	Yes
Hawkesworth et al. 2013 [24]	+	Unclear	Yes	Yes	Yes	Yes	Yes	Yes	Yes	Unclear	Yes	Yes	Yes	Yes	Yes
Observational Studies (*n* = 7)															
Goodyer et al. 2007 [32]	Ø	NA	Yes	Yes	NA	Yes	No	Unclear	No	Unclear	Yes	Yes	Yes	No	Yes
El-Khashab et al. 2013 [33]	Ø	NA	Yes	Yes	NA	Yes	Unclear	Unclear	Yes	Unclear	Yes	Yes	Yes	Yes	Unclear
Miliku, K. et al. 2017 [31]	+	NA	Yes	Yes	NA	Yes	Yes	Yes	Yes	Unclear	Yes	Yes	Yes	Yes	Yes
Miliku, K. et al. 2016 [30]	+	NA	Yes	Yes	NA	Yes	Yes	Yes	Yes	Unclear	Yes	Yes	Yes	Yes	Yes
Huang et al. 2014 [27]	Ø	NA	Yes	Yes	NA	Yes	Yes	Yes	Unclear	Unclear	Yes	No	Yes	Yes	Yes
Painter et al. 2005 [28]	+	NA	Yes	Yes	NA	Yes	Yes	Yes	No	Unclear	Yes	Yes	Yes	Yes	Yes
Miliku, K. et al. 2015 [29]	+	NA	Yes	Yes	NA	Yes	Yes	Yes	Yes	Unclear	Yes	Yes	Yes	Yes	Yes

[a] American Dietetic Association (ADA) Quality Criteria: Relevance questions (1) Would implementing the studied intervention or procedure (if found successful) result in improved outcomes for the patients/clients/population group? (NA for some Epi studies) (2) Did the authors study an outcome (dependent variable) or topic that the patients/clients/population group would care about? (3) Is the focus of the intervention or procedure (independent variable) or topic of study a common issue of concern to dietetics practice? (4) Is the intervention or procedure feasible? (NA for some epidemiological studies); Validity questions (1) Was the research question clearly stated? (2) Was the selection of study subjects/patients free from bias? (3) Were study groups comparable? (4) Was method of handling withdrawals described? (5) Was blinding used to prevent introduction of bias? (6) Were intervention/therapeutic regimens/exposure factor or procedure and any comparison(s) described in detail? Were intervening factors described? (7) Were outcomes clearly defined and the measurements valid and reliable? (8) Was the statistical analysis appropriate for the study design and type of outcome indicators? (9) Are conclusions supported by results with biases and limitations taken into consideration? (10) Is bias due to study's funding or sponsorship unlikely?

[b] Studies were rated as "positive" if they were assessed as a 'Yes' to all of the following four "essential" criteria, as follows; 1. Was selection of study subjects free from bias?; 2. Were study groups comparable?; 3. Were interventions and any comparisons described in detail?; 4. Were outcomes clearly defined and the measurements valid and reliable?; plus, at least one additional 'yes' from the other six criteria. If six or more of the criteria were assessed as a 'No', the study was rated "negative". Studies were rated "neutral" if the majority of the 10 criteria were met, but one of the "essential" criteria were not met.

NA: not applicable
